# High-grade appendiceal mucinous neoplasm: case report

**DOI:** 10.1093/jscr/rjag649

**Published:** 2026-07-30

**Authors:** Julio Caleb Arrieta Navarro, Julio Erick Leiva Valderrama, Sergio Daniel Coral Salvador, Nicole Karina Escudero Navarro, Alan Hari Silva Amaya, Sergio Dennys Aguilar Melin

**Affiliations:** Faculty of Human Medicine, National University of San Martín, Tarapoto, Peru; Faculty of Human Medicine, National University of San Martín, Tarapoto, Peru; Faculty of Human Medicine, National University of San Martín, Tarapoto, Peru; Faculty of Human Medicine, National University of San Martín, Tarapoto, Peru; Faculty of Human Medicine, National University of San Martín, Tarapoto, Peru; Department of Surgery and Specialties, Oncological Surgery Service, Hospital II-2 Tarapoto, Tarapoto, Peru; Faculty of Human Medicine, National University of San Martín, Tarapoto, Peru; Department of Medicine, Oncology Service, Hospital II-2 Tarapoto, Tarapoto, Peru

**Keywords:** mucinous adenocarcinoma, appendix, adnexal mass, hemicolectomy, case report

## Abstract

High-grade appendiceal mucinous neoplasm is an extremely rare gastrointestinal tumour with nonspecific clinical manifestations that complicate preoperative diagnosis. In women, it may mimic gynecological diseases, particularly ovarian tumours, leading to diagnostic delays and inappropriate surgical approaches. We report the case of a 68-year-old woman from the San Martín region of Peru who presented with abdominal pain, progressive distension, and abnormal vaginal bleeding. Laboratory tests showed elevated carcinoembryonic antigen levels, while transvaginal ultrasound revealed a right adnexal mass suggestive of ovarian neoplasia. During surgery, a mucinous appendiceal mass without ovarian involvement was incidentally identified, prompting modification of the surgical plan to an oncological right hemicolectomy. Histopathological analysis confirmed high-grade mucinous adenocarcinoma of the appendix. The postoperative course was favorable. This case highlights the importance of considering appendiceal neoplasms in the differential diagnosis of pelvic masses in postmenopausal women and the value of intraoperative and histopathological evaluation.

## Introduction

High-grade appendiceal mucinous adenocarcinoma (HAMN) is a rare epithelial neoplasm, representing <0.5% of gastrointestinal tumours and ~0.2%–0.3% of appendectomies [1, 2]. The global incidence of appendiceal mucinous neoplasms ranges from 0.12 to 2 cases per million inhabitants per year, of which only 10%–15% are HAMN [1, 3]. In Latin America and Peru, there are no robust epidemiological registries that would allow for estimating its true frequency [4].

Histopathologically, HAMN is characterized by mucinous epithelial proliferation with marked cytological atypia, complex architecture, and high histological grade, differentiating it from Low-grade appendiceal mucinous neoplasm (LAMN) and infiltrating adenocarcinoma [5]. Its clinical presentation is often non-specific, mimicking acute appendicitis, adnexal masses, or gynecological tumours, which complicates preoperative diagnosis [6]. In advanced stages, it can be associated with pseudomyxoma peritonei, characterized by the progressive accumulation of intra-abdominal mucinous material [7].

Definitive diagnosis requires histopathological and immunohistochemical examination. Treatment depends on the extent of the tumour: in localized disease, oncological right hemicolectomy is recommended, while in cases with peritoneal dissemination, the standard management includes cytoreductive surgery plus hyperthermic intraperitoneal chemotherapy, with a 5-year survival rate exceeding 60% in selected patients [7, 8]. However, access to these therapies remains limited in countries like Peru [4].

This report describes a case of mucinous cholecystoplasmic reticulum diagnosed incidentally during gynecological surgery, highlighting the importance of differential diagnosis between adnexal and appendiceal pathology, as well as the importance of histopathological examination to define the definitive surgical management.

## Clinical case

A 68-year-old woman, a homemaker residing in Las Palmas, Banda de Shilcayo district, San Martín region, Peru, with no personal or family history of cancer, harmful habits, or chronic medication, and with a complete vaccination schedule for her age.

The clinical picture began months earlier with non-specific abdominal pain and progressive abdominal distension, associated with an isolated episode of abnormal vaginal bleeding lasting 20 days. She subsequently experienced weight loss, decreased appetite, and nausea, prompting her to seek care at the Gynecology Department of Hospital.

On physical examination, she presented with superficial abdominal tenderness in the hypogastrium and flanks, as well as mild abdominal distension, without signs of peritoneal irritation or palpable masses. The gynecological examination initially suggested adnexal pathology, with no clinical evidence of gastrointestinal origin. Tumour markers showed elevated CEA (10.5 ng/ml), with normal CA-125 (11.9 U/ml) and alpha-fetoprotein (2.8 ng/ml). A transvaginal ultrasound revealed a right adnexal mass suggestive of an ovarian tumour. Given the suspicion of a Krukenberg tumour, a gastroenterology consultation was requested; however, an upper gastrointestinal endoscopy ruled out gastric malignancy.

With a presumptive diagnosis of ovarian neoplasia, surgical management was indicated. Preoperative tests showed elevated creatinine (1.89 mg/dl) and serum urea (50.79 mg/dl), which initially limited the use of contrast studies. Urinalysis revealed pyuria (40–45 leukocytes/high-power field). During hospitalization, mild renal insufficiency, urinary tract infection due to *Enterobacter cloacae*, and hypertension were diagnosed; these conditions were treated and stabilized before surgery.

Subsequently, computed tomography (CT) revealed a calcified chemical lesion on the anterior margin of the uterine fundus (131 × 77 × 44 mm) (Fig. 1), while magnetic resonance imaging (MRI) showed a calcified ovoid chemical lesion adjacent to the uterine fundus (61 × 49 × 132 mm), without infiltration of adjacent organs or lymphadenopathy (Fig. 2).

**Figure 1:**
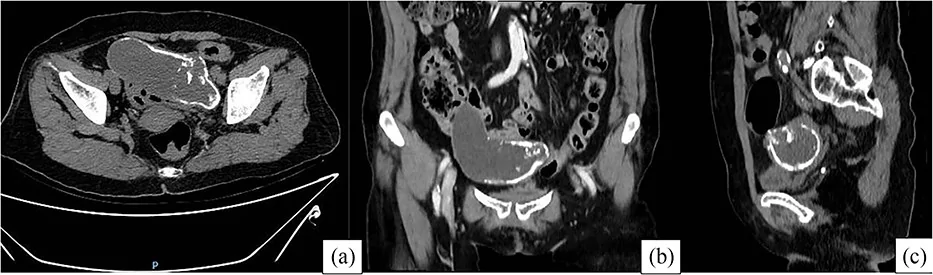
Contrast-enhanced CT scan of the pelvis. (a) Axial view showing a well-defined pelvic chemical lesion with thickly calcified walls, located in relation to the uterine fundus. (b) Coronal reconstruction showing an oval chemical mass ~13.1 × 7.7 × 4.4 cm, with extensive peripheral calcifications and homogeneous, low-attenuation contents, without significant enhancement after intravenous contrast administration. (c) Sagittal reconstruction confirming the pelvic location of the lesion and its relationship to the uterus, with no evidence of invasion of adjacent structures. Ascites is not observed.

**Figure 2:**
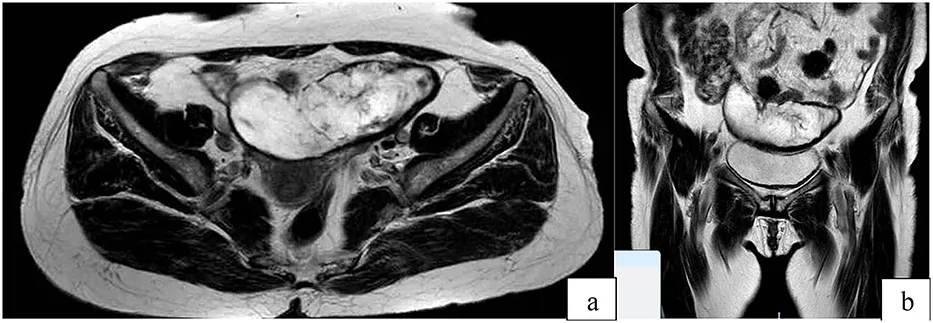
Thoraco-abdomino-pelvic MRI with contrast. (a) Axial and (b) coronal images showing a well-defined ovoid cystic pelvic mass, adjacent to the uterine fundus, measuring ~13.2 × 6.1 × 4.9 cm. No signs of local organ invasion, pelvic lymphadenopathy, or intraperitoneal free fluid are observed.

The patient was admitted with a presumptive diagnosis of bilateral ovarian neoplasia and was scheduled for exploratory laparotomy, bilateral salpingo-oophorectomy, total abdominal hysterectomy, and partial omentectomy. During surgery, a mucinous mass originating from the appendix was incidentally identified, while the ovaries and uterus appeared macroscopically normal. Given this finding, a right oncologic hemicolectomy with en bloc resection of the appendix, cecum, and proximal ascending colon was performed. The surgical specimen measured 18 × 8 × 5 cm (Fig. 3).

**Figure 3:**
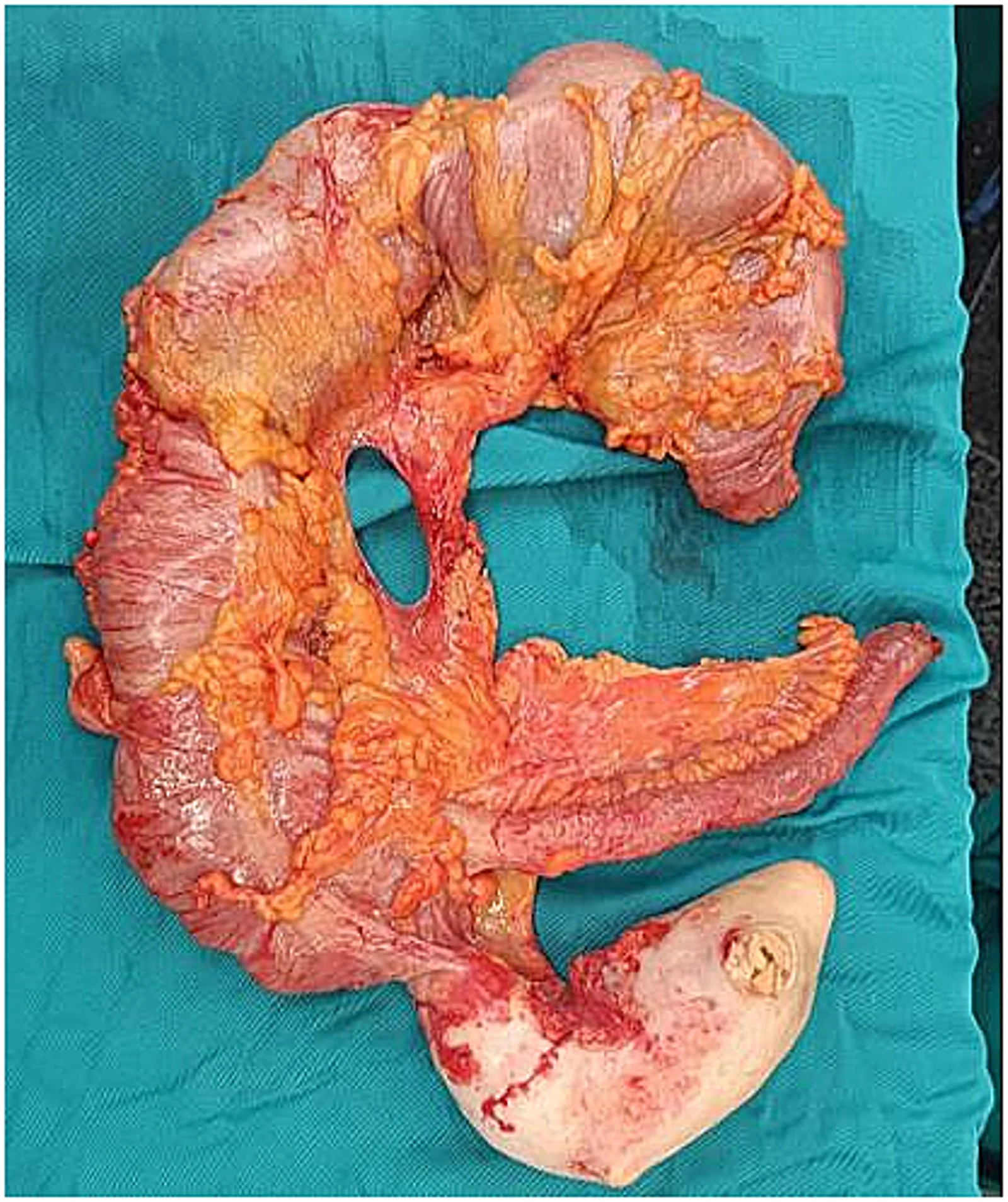
Images macroscopic post-surgical intervention. Right hemicolectomy specimen including markedly dilated cecal appendix, cystic and mucinous in appearance, compatible with appendiceal mucinous neoplasm.

The histopathological study confirmed a high-grade appendiceal mucinous neoplasm, with marked cytological atypia and architectural complexity, without primary ovarian involvement (Fig. 4). The postoperative course was favorable, with improvement of abdominal symptoms and hospital discharge for oncological follow-up and evaluation of adjuvant treatment according to the final staging.

**Figure 4:**
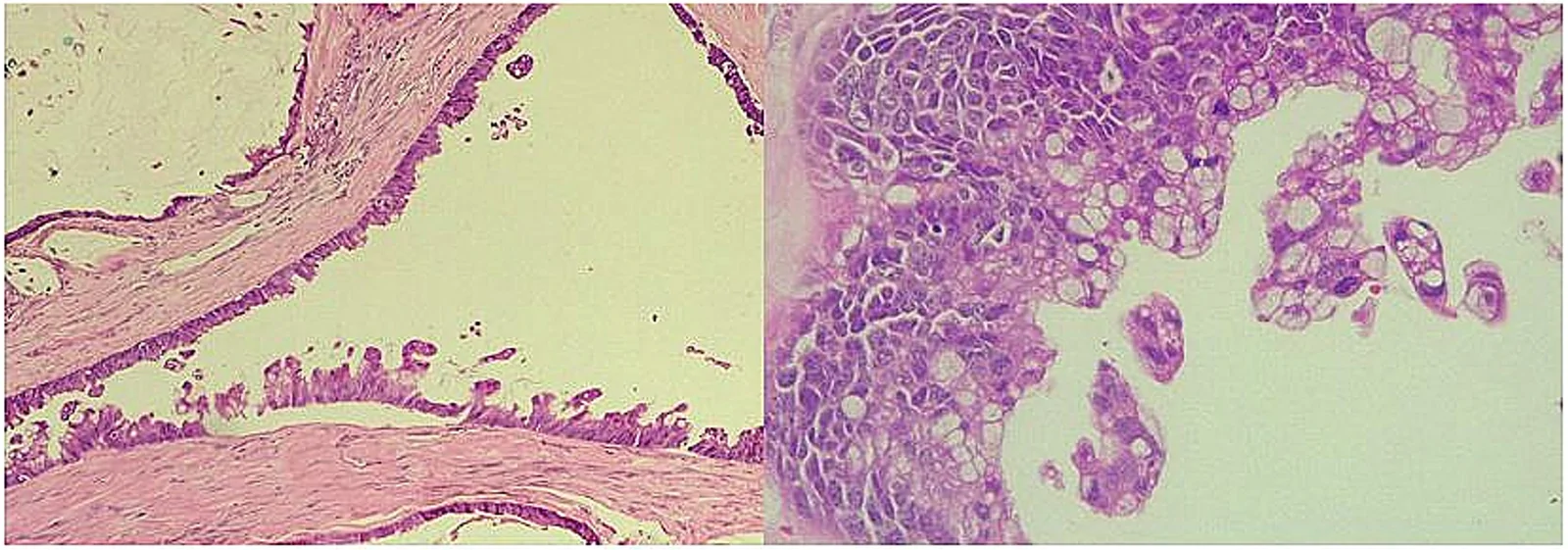
Microscopic images. Histological sections show an appendix with marked luminal dilation filled with abundant extracellular mucin. The mucosa shows mucinous epithelial proliferation with a complex papillary and villous architecture, lined by mucin-secreting columnar epithelium with abundant goblet cells, evidencing nuclear stratification, moderate to marked cytological atypia, and architectural disorganization.

## Discussion

Appendiceal neoplasms are rare and heterogeneous tumours, with an approximate annual incidence of six cases per million inhabitants [9]. They include epithelial and non-epithelial tumours of varying degrees of malignancy and are often confused with more common abdominal neoplasms, such as primary colorectal and ovarian tumours [10], which complicate their preoperative diagnosis.

They are primarily classified as adenocarcinomas and mucinous or non-mucinous neoplasms, the differences of which lie mainly in their histopathological characteristics and malignant potential (Table 1) [9, 11].

**Table 1:** Primary tumours of the appendix

PSOGI terminology	Characteristics	Other terminology
LAMN	Dysplastic lesion with low-grade cytology^*^, associated with architectural alterations such as loss of the lamina propria and muscularis mucosae, submucosal fibrosis, absence of infiltrative invasion^**^, with or without push invasion, mucin dissection in the wall, mucin or cells outside the appendix, and appendiceal rupture.	Mucinous tumour of uncertain malignant potential; mucinous tumor of low malignant potential
HAMN	Similar to LAMN, but with the presence of high-grade cytological features.	Non-invasive mucinous adenocarcinoma
Mucinous adenocarcinoma	Presence of infiltrative invasion^**^ by neoplastic epithelial cells.	Mucinous adenocarcinoma (AJCC grade 2 or 3)

Peritoneal Surface Oncology Group International (PSOGI) classification and terminology of primary appendiceal mucinous tumors, including histopathological characteristics and corresponding alternative nomenclature. LAMN: Low-grade appendiceal mucinous neoplasm; HAMN: High-grade appendiceal mucinous neoplasm; AJCC: American Joint Committee on Cancer.

High-grade mucinous appendiceal neoplasm is less frequent than low-grade mucinous neoplasm and appendiceal adenocarcinomas [12]. Its etiology is not fully understood, and due to its low incidence, the available evidence comes mainly from case reports and retrospective studies. Histopathologically, it is characterized by high-grade mucinous epithelial proliferation, with a risk of peritoneal dissemination and pseudomyxoma peritonei in advanced stages [12, 13].

Postoperative diagnosis, as in this case, is the most frequent way of identifying mucinous mucinous neoplasms, due to their nonspecific clinical presentation [14]. In this patient, vaginal bleeding and the adnexal mass observed by ultrasound initially suggested a gynecological pathology.

Although the findings suggested ovarian neoplasia, there were limitations in confirming it as a primary tumour. Elevated CEA levels may suggest appendiceal mucinous neoplasia, although they also increase in ovarian mucinous tumours and other gastrointestinal neoplasms [10, 15]. Similarly, CA-125 and CA19–9 lack specificity, as they can be elevated in response to peritoneal irritation or any mucinous tumour [10, 15].

The low clinical and biochemical specificity necessitated ruling out gastric tumours using upper gastrointestinal endoscopy. In women, HAMN frequently mimic adnexal masses due to their anatomical location and cystic or mucinous appearance on imaging studies [14]. Although CT and MRI are useful for initial evaluation, definitive diagnosis requires surgical resection and histopathological examination. Radiological findings such as local invasion may suggest high-grade lesions, but most diagnoses continue to be made by histopathology [13].

Exploratory laparotomy allowed for the identification of the appendiceal tumour and a modification of the initially gynecological surgical approach to an oncological right hemicolectomy, in accordance with current recommendations for localized, high-grade appendiceal neoplasms [9, 12]. In cases with peritoneal dissemination, cytoreductive surgery combined with hyperthermic intraperitoneal chemotherapy represents the most appropriate therapeutic strategy [15], although its availability is limited in middle-income countries.

The histopathological study confirmed the typical characteristics of a high-grade mucinous appendiceal neoplasm and ruled out primary ovarian involvement, a relevant finding due to the differences in management, prognosis, and follow-up between the two entities. Bilateral ovarian involvement, peritoneal dissemination, and a macroscopically abnormal appendix are frequently associated with mucinous appendiceal neoplasms [15]. Furthermore, oncological follow-up is essential, as these lesions can progress or coexist with appendiceal adenocarcinoma even after complete resection [11].

This case highlights the importance of considering a gastrointestinal origin in cases of mucinous adnexal masses, especially with elevated CEA levels. It also demonstrates the value of incidental intraoperative findings and the need to maintain a high index of suspicion for rare neoplasms or atypical presentations. Likewise, it underscores the relevance of histopathological examination to differentiate appendiceal cancer from other malignancies and to appropriately guide follow-up and treatment.

Limitations of this report include its descriptive nature and the lack of long-term follow-up. However, it provides local evidence on an extremely rare neoplasm and emphasizes the importance of thorough intraoperative exploration and histopathological analysis in therapeutic decision-making.

## Patient perspective

The patient expressed relief after the definitive diagnosis and positively assessed the surgical resolution and the established oncological follow-up, reporting clinical improvement after the intervention.
